# Impact of smoking on protein levels of beta-defensins in periodontal disease

**DOI:** 10.1590/0103-6440202204685

**Published:** 2022-08-26

**Authors:** Kahena Rodrigues Soldati, Lorena Silva Gutierrez, Giovana Anovazzi, Raquel Mantuaneli Scarel-Caminaga, Daniela Leal Zandim-Barcelos

**Affiliations:** 1 Department of Diagnosis and Surgery, São Paulo State University (Unesp), School of Dentistry, Araraquara, SP, Brazil.; 2 Department of Morphology, Genetics, Orthodontics and Pediatric Dentistry, São Paulo State University (Unesp), School of Dentistry, Araraquara, SP, Brazil.

**Keywords:** beta-defensins, periodontitis, smoking

## Abstract

Antimicrobial peptides (AMPs) are important components of the host response against invading pathogens. In addition to their direct antimicrobial activity, they can also participate in the immune system modulation. However, the role of AMPs in the etiopathogenesis of periodontal disease and the risk factors that may influence their expression in the oral cavity are not fully understood. The aim of this study was to determine the impact of smoking on beta-defensin (hBD) 1 and 2 levels analyzing samples from periodontitis patients. Fifty patients with periodontitis, 25 smokers and 25 non-smokers, and 20 periodontally healthy patients were recruited. After periodontal clinical evaluation, gingival crevicular fluid (GCF) samples were collected from healthy sites of patients without periodontal disease and from healthy and diseased sites of patients with periodontitis. Peptides quantification was performed by sandwich ELISA technique. Smokers showed reduced GCF hBD 1 levels and increased hBD 2 levels compared to non-smokers in diseased sites (p <0.05). Higher levels of hBD 1 were observed in healthy sites of patients without periodontal disease than in healthy sites of patients with periodontitis (p<0.0001). Diseased sites of non-smokers presented higher levels of hBD 2 than healthy sites (p <0.05). These results reveal that protein levels of hBDs 1 and 2 can be impaired by cigarette smoking in the presence of periodontal disease.

## Resumo

Peptídeos antimicrobianos (PAMs) são componentes importantes da resposta do hospedeiro contra patógenos invasores. Além de sua atividade antimicrobiana direta, eles também podem participar da modulação do sistema imunológico. No entanto, o papel dos PAMs na etiopatogenia da doença periodontal e os fatores de risco que podem influenciar a sua expressão na cavidade oral não são totalmente compreendidos. O objetivo deste estudo foi determinar o impacto do tabagismo nos níveis de beta-defensina (hBD) 1 e 2 analisando amostras de pacientes com periodontite. Cinquenta pacientes com periodontite, 25 fumantes e 25 não fumantes e 20 pacientes periodontalmente saudáveis foram recrutados. Após avaliação clínica periodontal, amostras de fluido crevicular gengival (FCG) foram coletadas de sítios saudáveis de pacientes sem doença periodontal e de sítios saudáveis e doentes de pacientes com periodontite. A quantificação dos peptídeos foi realizada pela técnica de ELISA sanduíche. Fumantes apresentaram níveis reduzidos de hBD 1 no FCG e níveis aumentados de hBD 2 em comparação com não fumantes em locais doentes (p <0,05). Níveis mais elevados de hBD 1 foram observados em sítios saudáveis de pacientes sem doença periodontal do que em sítios saudáveis de pacientes com periodontite (p<0,0001). Os sítios doentes de não fumantes apresentaram níveis mais elevados de hBD 2 do que os sítios saudáveis (p<0,05). Esses resultados revelam que os níveis das hBDs 1 e 2 podem ser prejudicados pelo tabagismo na presença de doença periodontal.

## Introduction

Periodontal disease is an inflammatory disorder resulted from the oral homeostasis disruption. The imbalance between the pathogenic potential of the biofilm and the host immune response can be especially compromised by some risk factors ^1^. It has been demonstrated that smokers are about three times more likely to develop periodontitis than non-smokers. In addition, smokers present greater attachment loss, rapid progression of periodontal destruction and less gingival bleeding than non-smokers ^2^.

Smoking is responsible for suppressing the immune response of periodontal tissue. It can decrease blood flow, impair neutrophil and monocytes activities, alters adhesion molecule expression and antibody production, as well as cytokine and inflammatory mediator release ^3^ ^,^ ^4^. Additionally, smoking inhibits oral tissue proliferation, inhibits attachment and migration of fibroblasts and promotes the differentiation of osteoclasts in periodontal tissue ^2^. Nevertheless, the effect of smoking on antimicrobial peptides (AMPs) production and release is still not completely understood.

Intended to maintain a healthy balance, AMPs, such as human beta-defensins (hBDs), can be synthesized by a variety of epithelial cells. In the oral cavity, they are present in gingival tissue, tongue, salivary glands and mucosa, gingival crevicular fluid (GCF) and saliva ^5^. AMPs have special properties that allow them to act directly against bacterial, viral and fungal invasion ^6^. They can bind to microbial membranes through electrostatic and hydrophobic interactions, resulting in elimination of the microorganisms by disrupting their cell membranes ^7^. Besides its direct killing activity, AMPs can promote immunomodulatory activities, being chemoattractive for effector cells and stimulating the production and release of various immunoregulatory mediators by inflammatory cells. Therefore, they are recognized as potent agents in inflammatory processes and modulators of adaptive immunity ^8^.

Few in vitro studies have demonstrated that smoking can affect the expression of AMPs in gingival epithelial cells or skin keratinocytes. In the nicotine pre-treated HaCaT culture, followed by TNF-α stimulus, it was reported a reduction of hBD 2 expression when compared to the control group. A similar result was observed in *Porphyromonas gingivalis (Pg)* lipopolysaccharide (LPS)-stimulated epithelial cells treated with cigarette smoke extract ^9^. On the other hand, cigarette smoke extract promoted a significant decrease in hBD 1 expression, but increased the expression of hBD 2 in human epithelial cells. In a previous clinical study, the expression of hBD 1 and 2 genes was down-regulated in non-inflamed gingival samples of smokers compared to non-smokers ^10^. Nevertheless, a higher GCF hBD 2 level was observed in smokers than in non-smokers with periodontitis ^11^. Therefore, the smoking mechanisms involved in AMPs regulation need to be more elucidated.

Considering that AMPs produced by epithelial cells appear to play an important role in the innate immune defense system of the oral cavity, the present study was performed to evaluate the possible impact of smoking on protein levels of human hBD 1 and 2. We hypothesized that the GCF levels of hBD 1 and 2 can be negatively affected by smoking. The GCF hBD 1 and 2 levels in healthy and diseased sites of smokers and non-smokers with periodontitis was compared.

## Material and methods

### Study design

The study protocol was approved by the Ethics Committee on Human Research of the School of Dentistry at Araraquara - UNESP (protocol number 01394712.5.0000.5416). The purpose and content of the study were explained to eligible subjects, and written informed consent was obtained from all participants for inclusion in the study.

A total of 70 participants were recruited from June 2016 to March 2018 at the Periodontology Clinic, School of Dentistry at Araraquara - UNESP. The diagnosis of periodontitis was performed according to the classification of the 2017 World Workshop on the Classification of Periodontal and Peri-implant Diseases and Conditions ^12^. All patients should present a minimum of 15 natural teeth. Twenty patients were included in the periodontally healthy control group. All subjects were free of systemic and periodontal diseases. They should present probing depth (PD) ≤ 3 mm, no bleeding on probing (BOP), no clinical attachment loss, plaque index (PI) and gingival index (GI) ≤ 15%. Fifty patients with periodontitis stage III or IV were included in periodontitis groups. In addition to the diagnostic criteria ^12^, they should present at least five sites in non-adjacent teeth with PD ≥ 4 mm, BOP, and clinical attachment level (CAL) ≥ 4 mm, except for third molars. From the 50 patients, 25 had never smoked and 25 were smokers who should smoke at least 10 cigarettes/day for a minimum of 5 years.

None of the patients had a history of systemic diseases or immunological disorders. Subjects who had received antibiotics and anti-inflammatory drugs within the last 3 months or received periodontal treatment within the last 6 months were not included in the study. Patients using hormone replacement therapy, pregnant and lactating women and orthodontic patients were also excluded.

The sample size was calculated using statistical software (GPower 3.1 Statistical Power Analysis for Windows, Düsseldorf, Germany). Considering mean values of hBD expression and standard deviation from previous studies ^13^ ^,^ ^14^ ^,^ ^15^, an effect size of 0.3 was obtained. Based on this effect size, a significance level of 0.05 and a study power of 0.80, a sample size of 20 individuals per group were determined to be necessary.

### Clinical examination

One calibrated examiner performed all clinical examinations. The intra-examiner reproducibility was assessed by measuring the PD in six teeth (16, 21, 24, 36, 41 and 44), according to the Ramfjord index, in a total of six patients (36 sites/patient) on two different occasions in a time interval of 7 days. Data were submitted to Kappa concordance analysis (k=0.91). The parameters PD, BOP and CAL were assessed at six sites per tooth (mesio-buccal, mid-buccal, disto-buccal, mesio-lingual, mid-lingual, disto-lingual), while PI and GI were registered as absence (negative site) or presence (positive site) of visible plaque and gingival bleeding at four sites per tooth (mesial, distal, buccal and lingual), excluding the third molars. Measurements were performed with a manual periodontal probe (UNC15; Hu-Friedy, Chicago, IL, USA).

### Gingival crevicular fluid (GCF) sampling

GCF samples were collected from healthy (n=5) sites of each periodontally healthy patient and from healthy (n=5) and diseased (n=5) sites of each periodontitis patient. Sites at different non-adjacent teeth, preferably one site per quadrant, were selected for GCF collection in each subject, one day after the periodontal examination. Healthy sites had no gingival inflammation, PD ≤ 3 mm, no BOP or clinical attachment loss; and diseased sites had gingival inflammation, PD and CAL ≥ 4 mm with BOP. Diseased sites were selected according to the deepest probing depth in each quadrant.

A single examiner performed GCF sampling. The sites to be sampled were isolated with cotton rolls, the supragingival plaque was removed with a sterile curette and the surfaces were gently air-dried. GCF was sampled by inserting absorbent paper strips (Periopaper^®^, Oraflow Inc., New York, USA) into the gingival sulcus or periodontal pocket for 30 seconds. Paper strips contaminated with blood and saliva were rejected. An electronic device Periotron 8000 determined the GCF volume absorbed into each strip (Periopaper, ProFlow, Inc., Amityville, NY, USA), which was calibrated based on a protocol described before ^16^ (calibration of the electronic device by polynomial regression). The GCF was obtained as a pooled sample of the same category (healthy or diseased site) in each subject. The papers strips were immediately placed into a dry sterile polypropylene tube (5 paper strips per tube) and kept at -80 °C until analysis. The readings from the Periotron 8000 were converted to an actual volume (μL) by reference to a standard curve.

### Immunosorbent assay for hBD 1 and hBD 2 quantification

First, the absorbed GCF was eluted from the paper strips by adding 750 μL (150 μL per strip) of 1x Phosphate-Buffered Saline (PBS) to each tube. The tubes were left on ice in a horizontal shaker for 30 minutes and then centrifuged at 4 °C, 13.000 g for 10 minutes. The GCF levels of hBD 1 and 2 were measured using the sandwich ELISA technique according to the manufacturer’s guidelines (Peprotech, Rocky Hill, NJ, USA). The absorbance was measured by a spectrophotometer at 450 nm wavelength. The levels of AMPs in each sample were determined using the concentration values of standards included in the kit contents. The results were expressed in pg/mL.

### Statistical analysis

The statistical analysis was performed using the software GraphPad Prism 7 (San Diego, CA, USA). For clinical data, normal distribution was analyzed using the D’Agostino and Pearson test. For normally distributed data, it was used One-way ANOVA followed by Tukey for healthy sites and unpaired t test for diseased sites on inter-group analyses and paired t test for intra-group analyses. For non-normally distributed data, Kruskal-Wallis and Dunn were used for healthy sites and Mann-Whitney tests was used for diseased sites on inter-group analyses, while the Wilcoxon test was performed for intra-group comparison. A p-value of less than 0.05 was considered significant. The effect size was estimated for the primary outcome of the study (AMPs level in GCF). Since non-parametric tests were used for intra and inter-group comparisons, the eta squared and the correlation coefficient r were calculated. ^17^.

## Results

### Demographics and periodontal parameters

A significant age difference was found between periodontally healthy subjects (25.79 ± 3.95) and periodontitis patients (NS [40.32 ± 12]; S [43.88 ± 10.64]) (p<0.05). The distribution of the male and female participants had no significant difference between the study groups, periodontally healthy subjects (5 males/15 females) and periodontitis patients (NS - 9 males/16 females; S - 12 males/13 females) (p = 0.28).

The periodontal clinical parameters are presented in table 1. Regarding the full mouth periodontal clinical parameters, PI, BOP, PD and CAL were significantly lower in periodontally healthy subjects than in periodontitis patients (p<0.0001). Only for GI, a significant difference was observed between periodontally healthy patients and smoker patients (p=0.0001).

**Table 1:** Full-mouth clinical periodontal parameters of the study groups. Parametric data are expressed as mean ± standard deviation (SD) and non-parametric data are expressed as median (25th; 75th percentiles).

Clinical Parameters	Healthy (n=20)	Non-smokers (n=25)	Smokers (n=25)
PI (%)	7.14 (1.78; 9.32)	29.62 ± 14.82	35.01 ± 21.77
GI (%)	1.78 (0.89; 2.67)	12.45 ± 7.72	10.25 ± 15.03
BOP (%)	2.98 (0.6; 5.95)	19.45 ± 14.73	21.89 ± 23.72
PD (mm)	1.49 ± 0.29^(^	2.92 ± 0.54	3.05 ± 0.72
PD ≤ 3mm (%)	100^*^	80 (65.5; 94.5)	83 (71; 89)
PD 4-5mm (%)	0^*^	17 (5.5; 27.5)	15 (10; 24)
PD ≥ 6mm (%)	0^*^	1 (0; 5)	1 (0.5; 5)
CAL (mm)	1.49 ± 0.29^(^	3.30 ± 0.99	3.47 ± 0.91
CAL ≤ 3mm (%)	100 (100; 100)	39 (17.5; 51.5)	29 (22.5; 43)
CAL 4-5mm (%)	0	45 (34; 49.5)	46 (32.5; 53.5)
CAL ≥ 5mm (%)	0^*^	17 (8; 31.5)	17 (9.5; 38)

* p <0.0001 - Significant difference from the other groups, Kruskal-Wallis with Dunn test; ^(^ p = 0.0001 - Significant difference from non-smokers, Kruskal-Wallis with Dunn test; ^(^ P <0.0001 - Significant difference from the other groups, One-way ANOVA with Tukey test. PI - Plaque index; GI - Gingival index; BOP - Bleeding on probing; PD - Probing depth; CAL - Clinical attachment level.

The sampled sites of periodontally healthy subjects presented significantly lower values of PD and CFV than healthy sampled sites of smokers and non-smokers with periodontitis (p<0.05), as well as lower CAL than healthy sampled sites of smokers (p<0.05) (Table 2). In patients with periodontitis, the intra-group comparison revealed lower levels of PD, CAL and CFV in the healthy sampled sites than in the diseased sites (p<0.0001) (Table 2).

**Table 2:** Clinical periodontal parameters of sampling sites. Data are given as median (25th; 75th percentiles).

Clinical Paramentes	Periodontally Healthy (n=20)	Non-smokers (n=25)		Smokers (n=25)	
	Healthy	Healthy	Diseased	Healthy	Diseased
PD (mm)	2.0 (1.0; 2.0)^a^	2.0 (1.83; 2.5)^A^	5.2 (4.5; 6.0)	2.0 (1.8; 2.4)^A^	5.0 (4.7; 6.5)
CAL (mm)	2.0 (1.0; 2.0)^a^	2.0 (2.0; 2.6)^A^	5.6 (4.5; 6.6)	2.4 (2.0; 2.9)^A^	5.4 (5.0; 7.0)
CFV ((L)	0.16 (0.12; 0.26)^a^	0.48 (0.28; 0.71)^A^	0.89 (0.61; 1.09)	0.58 (0.25; 0.78)^A^	0.86 (0.56; 1.16)

a p<0.05 - Significant difference from the other healthy groups, Kruskal-Wallis with Dunn test; A P <0.0001 - Significant difference from diseased sites of the same group, Wilcoxon test. PD - Probing depth; CAL - Clinical attachment level; CFV - Crevicular fluid volume

### hBD 1 and hBD 2 quantification

**Figure 1:**
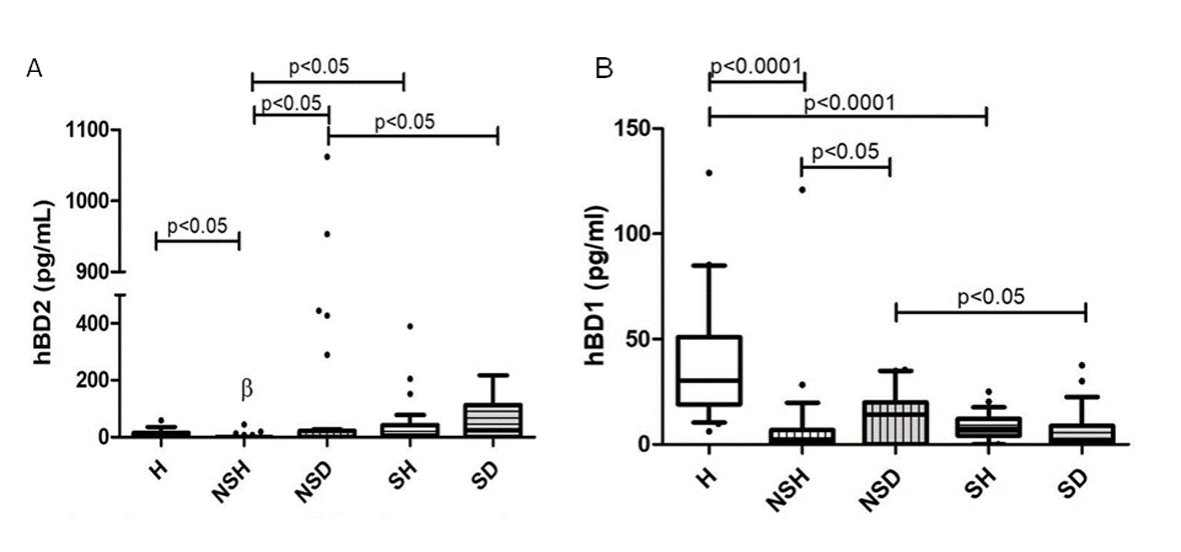
Distribution of antimicrobial peptides at the sites selected for GCF sampling. A) hBD 1 levels; B) hBD 2 levels. The results are shown as box plots with medians (lines inside boxes), 25th and 75th percentiles (limits of boxes) and the 10th and 90th percentiles (whiskers). H (n=20) - periodontally healthy group; NS (n=25) - non-smoker group: NSH - healthy sites of non-smokers and NSD - diseased sites of non-smokers; S (n=25) - smoker group: SH - healthy sites of smokers and SD - diseased sites of smokers.

The GCF levels of hBD 1 and hBD 2 are presented in Figure 1. The hBD 1 levels were significantly higher in healthy sites of the patients without periodontal disease than in healthy sites of smokers and non-smokers with periodontitis (p<0.0001), with a mean difference of 31.62 (95% CI: 17.15 - 46.08) between SH and H and a mean difference of 31.20 between NSH and H (95% CI: 13.81 - 48.57). The lowest GCF hBD 2 level was observed in healthy sites of non-smokers (p=0.0003). A mean difference of 33.61 was observed between SH and H (95% CI: -3.41 - 70.63), and a mean difference of 6.56 was found between NSH and H (95% CI: -1.68 - 14.79). The magnitude of the effect observed for GCF levels of hBDs in healthy sites was large (eta squared= 0.497 and r= 0.70 for hBD 1; eta squared= 0.176 and r= 0.42 for hBD 2) ^17^.

The comparison between diseased sites of patients with periodontitis revealed significantly higher levels of hBD 2 and lower levels of hBD 1 in the GCF of smokers compared to non-smokers (p<0.05). The mean difference between SD and NSD was 74.67 (95% CI: -50.82 - 200.16) for hBD 2 and 6.89 (95% CI: 0.64 - 13.14) for hBD 1. The magnitude of the effect observed for GCF levels of hBDs in diseased sites of smokers and non-smokers was intermediate (hBD 2 eta squared= 0.073 and r= 0.27; hBD 1 eta squared= 0.081 and r= 0.28) ^17^.

In non-smokers, higher levels of hBD 1 and 2 were observed in diseased sites than in healthy sites (p<0.05). A mean difference of 4.34 for hBD 1 (95% CI: -6.74 - 15.41) and 118.95 for hBD 2 (95% CI: -3.14 - 241.04) was observed between NSD and NSH. The magnitude of the effect observed was intermediate for hBD 2 (eta squared= 0.097 and r= 0.31) and small for hBD 1 (eta squared= 0.021 and r= 0.14). However, in smokers, no significant difference in hBDs level was found between healthy and diseased sites (p>0.05). A mean difference of 2.13 for hBD 1 (95% CI: -2.71 - 6.96) and 10.67 for hBD 2 (95% CI: -36.24 - 57.58) was observed between SD and SH. The magnitude of the effect observed was small for hBD 2 (eta squared= 0.019 and r= 0.14) and intermediate for hBD 1 (eta squared= 0.108 and r= 0.33) ^17^.

## Discussion

The clinical results of the present study showed that smoking has a significant effect on GCF hBD 1 and hBD 2 levels. Reduced GCF hBD1 levels were observed in diseased sites of smokers compared to the same sites of non-smokers. In healthy sites, no difference was found between hBD 1 levels in periodontitis patients regarding smoking status. However, higher levels of hBD 2 were detected on healthy and diseased sites of smokers compared to non-smokers. To the best of our knowledge, this is the first study to evaluate hBD 1 and 2 simultaneously among smoker patients with periodontitis. Ertugrul et al. ^11^ also demonstrated that smoker patients either with generalized aggressive periodontitis or with gingivitis presented higher GCF levels of hBD 2 than non-smoker patients. On the other hand, a previous study reported a significant reduction of hBD 1 and 2 gene expression in non-inflamed gingival samples of smokers compared to non-smokers ^10^. Evidence indicates that smoking is associated with oral microbiome modification in patients with periodontitis. Smokers showed a greater abundance of periodontopathogenic microorganisms in subgingival plaque than non-smokers ^18^. In addition, cytotoxic and immunosuppressive effects of smoking to the periodontal tissues have been described. It inhibits blood flow, oral tissue proliferation, attachment and migration of fibroblasts, affects neutrophil function and activates inflammatory cytokines ^4^ ^,^ ^5^ ^,^ ^6^ ^,^ ^7^. Generally, hBD 2 are expressed at low levels, possibly being induced in response to pathogens invasion and inflammation ^19^. The increased number of periodontopathogens in subgingival biofilm, the impaired neutrophils chemotaxis and phagocytosis, and the presence of inflammatory cytokines may explain the increased release of hBD 2 in GCF of smokers.

Conversely, higher levels of hBD 1 could be found in the GCF in absence of stimulus, which can present a constitutive activity ^20^. In the present study, a higher concentration of hBD 1 was observed in healthy sites of periodontally healthy subjects than in healthy sites of patients with periodontitis. This result suggests that the reduction of GCF hBD1 levels may be related to the periodontal status and not to the smoking habit. Costa et al. ^21^ also reported that significantly higher levels of hBD 1 in periodontally healthy patients compared to healthy sites of patients with periodontitis. A previous study suggested that genetic intrinsic properties may regulate the hBD inductive expression by epithelial cells ^22^. They reported that gingival epithelial cells presented similar regulatory pathways for hBDs in periodontitis and healthy samples in response to the same inflammatory stimulus. However, different outcomes were registered, periodontitis samples expressed lower levels of hBDs than healthy samples ^22^. Reinforcing these findings, Brancatisano et al. ^14^ ^)^ reported higher expression of hBD 3 in GCF of healthy patients in comparison with diseased sites of periodontitis patients. Although not expected, lower levels of hBD 2 were found in healthy sites of non-smokers than in healthy sites of patients without periodontal disease and smokers.

The comparison between healthy and diseased sites of patients with periodontitis revealed different results for non-smokers and smokers. The non-smoker group showed significantly higher GCF hBD 1 and 2 levels in diseased sites compared to healthy sites. Increased hBDs levels may have been released to the GCF from periodontal epithelial cells as a protection mechanism against the increase of specific microorganism colonization. Moreover, it is already known that hBD 2 is stimulated by the presence of pathogenic bacteria as well as inflammatory stimulus ^20^. On the other hand, our data showed no statistical difference on hBD 1 and hBD 2 levels between healthy and diseased sites of smokers. This result may support the assumption that cigarette compounds may be responsible for the modulation of AMP in periodontal disease. Several toxins present in cigarette and other forms of tobacco smoke have varying immunomodulatory effects, such as neutrophil migration, chemotaxis and phagocytosis and production of chemical mediator.

In fact, the mechanisms by which cigarette smoking exacerbates inflammation in chronic inflammatory diseases are not completed understood and the effect of smoking and its toxic products on AMP regulation should be more explored. Recently, Donate et al. ^23^ revealed a new mechanism by which smoking may exacerbate rheumatoid arthritis. The transcription of miR-132, activated by cigarette smoking-induced AhR activation in Th17 cells, enhanced osteoclastogenesis and contributed to the development and progression in experimental and clinical rheumatoid arthritis.

According to our inclusion criteria, the difference observed in clinical parameters between periodontally healthy and periodontitis patients was expected. The periodontal condition of patients in both groups with periodontitis was similar, allowing the evaluation of smoking habit effect on AMPs regulation without the interference of different periodontitis severity. Only the parameter GI was not statistically different between periodontally healthy patients and periodontitis smoker patients. GI is considered a sign of periodontal tissue inflammation. Nevertheless, it seems to be reduced in smokers. It was demonstrated in a human model of experimental gingivitis that the development of inflammation in response to bacterial biofilm accumulation is reduced in smokers when compared to non-smokers ^24^. According to Dietrich ^25^, smoking may have a chronic, dose-dependent effect on gingival bleeding suppression.

The present study has some limitations. The smoking status was defined based on self-report of smoking, no biochemical analysis was performed to confirm it. Moreover, the samples of healthy and diseased sites of smokers were derived from the same patient. The inclusion of a smoker group without periodontal disease may be interesting in further studies. Although the sample size was calculated, the effect size estimation for the primary outcome of the study showed an intermediate effect for most of the comparisons. Studies including a large sample size should be considered for a better understanding of the smoking effect on hBD 1 and 2 expression in patients with periodontitis.

In conclusion, smoking habit may interfere in the immune response by regulating the GCF levels of hBD 1 and hBD 2. Smokers presented reduced concentration of hBD 1 and increased concentration of hBD 2 in diseased sites than non-smokers. Thus, it is conceivable that the increased severity of periodontitis in smokers may be in part explained by the influence of tobacco products on hBDs expression. The knowledge of how risk factors, such as smoking, can impair hBDs functions, can bring some insights into how each peptide works, and how important are AMPs for the immune response.
